# Comparison of Bispectral Index™ values during the flotation restricted environmental stimulation technique and results for stage I sleep: a prospective pilot investigation

**DOI:** 10.1186/s13104-017-2947-4

**Published:** 2017-11-29

**Authors:** C. Michael Dunham, Jesse V. McClain, Amanda Burger

**Affiliations:** 1 Trauma, Critical Care, and General Surgery Services, St. Elizabeth Youngstown Hospital, 1044 Belmont Ave., Youngstown, OH 44501 USA; 2 Advanced Neurology Associates, Inc, 1340 Belmont Ave., Youngstown, OH 44504 USA; 3 Behavioral Medicine, St. Elizabeth Family Medicine Residency, 1053 Belmont Ave., Youngstown, OH 44504 USA

**Keywords:** Flotation-REST, Flotation-tank, Sensory isolation, Relaxation, Bispectral Index, BIS monitor, Depth of sleep, Perception of sleep, Sleep EEG

## Abstract

**Objective:**

To determine whether Bispectral Index™ values obtained during flotation-restricted environment stimulation technique have a similar profile in a single observation compared to literature-derived results found during sleep and other relaxation-induction interventions.

**Results:**

Bispectral Index™ values were as follows: awake-state, 96.6; float session-1, 84.3; float session-2, 82.3; relaxation-induction, 82.8; stage I sleep, 86.0; stage II sleep, 66.2; and stages III–IV sleep, 45.1. Awake-state values differed from float session-1 (%difference 12.7%; Cohen’s d = 3.6) and float session-2 (%difference 14.8%; Cohen’s d = 4.6). Relaxation-induction values were similar to float session-1 (%difference 1.8%; Cohen’s d = 0.3) and float session-2 (%difference 0.5%; Cohen’s d = 0.1). Stage I sleep values were similar to float session-1 (%difference 1.9%; Cohen’s d = 0.4) and float session-2 (%difference 4.3%; Cohen’s d = 1.0). Stage II sleep values differed from float session-1 (%difference 21.5%; Cohen’s d = 4.3) and float session-2 (%difference 19.6%; Cohen’s d = 4.0). Stages III–IV sleep values differed from float session-1 (%difference 46.5%; Cohen’s d = 5.6) and float session-2 (%difference 45.2%; Cohen’s d = 5.4). Bispectral Index™ values during flotation were comparable to those found in stage I sleep and nadir values described with other relaxation-induction techniques.

**Electronic supplementary material:**

The online version of this article (10.1186/s13104-017-2947-4) contains supplementary material, which is available to authorized users.

## Introduction

The flotation-restricted environmental stimulation technique (REST), a process of floating horizontally and face up inside a quiet, dark tank filled with magnesium sulfate-saturated water held at normal skin temperature, has been associated with multiple benefits [1, 2]. However, investigations assessing intracranial neurophysiological events are limited. Sakata et al. showed that during float sessions stage I and stage II sleep were each present [3]. Using electroencephalography (EEG) monitoring, investigators have demonstrated increases in delta power [3] and theta power [4], findings also documented during sleep [5]. The only other flotation EEG investigations have not provided spectral power analysis information [6–8].

Since the first author has had experience with Bispectral Index™ (BIS) monitoring in the intensive care environment [9, 10] and is conducting an investigation using the BIS monitor as a tool for neurofeedback (Clinical Trials.gov: NCT03152331), we became interested in performing BIS monitoring during flotation-REST sessions. BIS values have been associated with distinct sleep stages [11–15], and found to decrease with relaxing guided imagery [16] and when viewing calming videos [17]. We hypothesized that BIS values obtained during flotation-REST would have a similar profile compared to literature-derived results found during sleep and other relaxation-induction interventions.

## Main text

### BIS monitoring during flotation

From April 7, 2017 to July 14, 2017, the first author underwent 22 1-hour flotation-REST sessions. During sessions 14 and 16, BIS monitoring was performed where BIS values were recorded on the BIS-X hard drive every minute during flotation. The monitor used was the BIS VISTA™ monitoring system, and the sensor established cutaneous contact at the FPz, FP2, AF8, and FT10 positions. As the monitor was located external to the tank and the first author was floating in darkness, there was no visual perception of the evolving BIS values. BIS values recorded on the hard drive were entered into a Microsoft Excel^®^ 2010 spreadsheet and imported into SAS System for windows, release 9.2 for statistical analyses.

### Literature-derived BIS values

A PubMed literature search was performed to identify manuscripts that provided BIS values obtained during the awake state. An overall weighted mean BIS value in the awake state was computed after entering the mean value for each cohort into the Excel spreadsheet according to the number of individuals participating in the cohort investigation. Also, we identified manuscripts that provided BIS values obtained during sleep monitoring. An overall weighted mean BIS value was computed for each sleep stage. An overall weighted mean BIS value was also computed for the two relaxation-induction studies.

### Mood state assessment

Pre-flotation and post-flotation session mood scores were recorded for each session [18]. Using the mood qualities relaxation and freshness, two orientating prompts “right now I feel relaxed” and “right now I feel fresh” were evaluated. Each mood quality was scored as follows: definitely not, 1; not, 2; not really, 3; a little, 4; very much, 5; and extremely, 6. The two mood scores were summed (range 2–12).

### Statistical analysis

A two-sample t test was used to compare intergroup BIS values and determine significant differences (*p* < 0.05). Cohen’s d statistic was computed for intergroup BIS value differences. Pre-flotation and post-flotation mood scores were compared using the Wilcoxon test for paired samples and Cohen’s d statistic.

## Results

BIS values for awake patients emanated from 20 cohorts within 18 studies [14, 16, 19–34] (Additional file 1).

Float session-1 monitoring lasted for 64 min and resulted in a BIS value of 84.3 ± 4.5 (range 77–98). The BIS value at 2 min was 83. The duration of float session-2 was 63 min with a BIS value of 82.3 ± 4.1 (range 77–97). The BIS value at 3 min was 83.

BIS values for stage I sleep emanated from 42 participants in 3 studies (Table 1). BIS values for stage II sleep came from 25 participants in 2 studies (Table 1). BIS values for stages III–IV sleep emanated from 39 participants in 4 studies (Table 1). The weighted-mean BIS progressively decreased from sleep stage I to sleep stages III–IV.

**Table 1:** BIS values by sleep stage

Sleep stage	Participants	BIS
Stage I		
Benini [11] {#2}	15	88.9
Dahaba [12] {#4}	10	80.0
Tung [13] {#35}	17	86.5
	*42*	*86.0 ± 3.5*
Stage II		
Benini [11] {#2}	15	63.0
Dahaba [12] {#4}	10	71.0
	*25*	*66.2 ± 4.0*
Stage III–IV		
Benini [11] {#2}	3	33.0
Benini [11] {#2}	1	16.0
Dahaba [12] {#4}	10	52.0
Dahaba [12] {#4}	10	41.0
Nieuwenhuijs [14] {#25}	10	42.0
Sleigh [15] {#33}	5	59.0
	*39*	*45.1 ± 8.9*

*BIS* Bispectral Index™

Nadir BIS values during relaxation-induction emanated from 54 participants in 2 studies [16, 17] (Additional file 2).

BIS value comparisons for the various conditions are presented in Table 2. The minimal mean percent differences and low Cohen’s d values indicate that float session-1 and float session-2 were similar to relaxation induction and sleep stage I. The other comparisons demonstrated substantial mean differences between float session-1 and float session-2 and the awake state, stage II sleep, and stages III–IV sleep. The awake state and each sleep stage had a relatively large BIS mean difference (*p* < 0.0001), and the Cohen’s d values were as follows: awake state versus stage I sleep, 3.9; stage I versus stage II sleep, 5.3; and stage II versus stages III–IV sleep, 3.1.

**Table 2:** Comparisons of BIS data by clinical conditions

	BIS	*p* value	%difference	Cohen’s d
Awake state	96.6 ± 1.7			
Float session-1	84.3 ± 4.5	< 0.0001	12.7	3.6
Awake state	96.6 ± 1.7			
Float session-2	82.3 ± 4.1	< 0.0001	14.8	4.6
Relaxation induction	82.8 ± 6.8			
Float session-1	84.3 ± 4.5	0.1548	1.8	0.3
Relaxation induction	82.8 ± 6.8			
Float session-2	82.3 ± 4.1	0.6256	0.5	0.1
Sleep stage I	86.0 ± 3.5			
Float session-1	84.3 ± 4.5	0.0292	1.9	0.4
Sleep stage I	86.0 ± 3.5			
Float session-2	82.3 ± 4.1	< 0.0001	4.3	1.0
Float session-1	84.3 ± 4.5			
Sleep stage II	66.2 ± 4.0	< 0.0001	21.5	4.3
Float session-2	82.3 ± 4.1			
Sleep stage II	66.2 ± 4.0	< 0.0001	19.6	4.0
Float session-1	84.3 ± 4.5			
Sleep stages III–IV	45.1 ± 8.9	< 0.0001	46.5	5.6
Float session-2	82.3 ± 4.1			
Sleep stages III–IV	45.1 ± 8.9	< 0.0001	45.2	5.4

*BIS* Bispectral Index™

Pre-flotation and post-flotation mood scores are depicted in Table 3. The mean pre-flotation mood score was 6.5 ± 1.3 (median 6.0), and the mean post-flotation mood score was 10.0 ± 1.6 (median 10.0) (*p* < 0.0001, Wilcoxon test for paired samples [two-tailed]; Cohen’s d = 2.4). Pre-flotation mood scores progressively increased from 5 at sessions 1–7 to 8 at sessions 16–22. Similarly, post-flotation mood scores increased during later sessions. The mean pre-flotation and post-flotation difference for the 22 float sessions was 3.5 ± 0.5.

**Table 3:** Pre-flotation and post-flotation mood scores by flotation sessions

Session	Pre-flotation	Post-flotation	Difference	BIS monitoring
1	5	8	3	
2	5	8	3	
3	5	8	3	
4	5	8	3	
5	5	8	3	
6	5	8	3	
7	5	8	3	
8	6	9	3	
9	6	10	4	
10	6	10	4	
11	6	10	4	
12	6	10	4	
13	7	10	3	
14	7	10	3	Session-1
15	7	10	3	
16	8	12	4	Session-2
17	8	12	4	
18	8	12	4	
19	8	12	4	
20	8	12	4	
21	8	12	4	
22	8	12	4	

*BIS* Bispectral Index™

## Discussion

### EEG spectral power and BIS for flotation-REST and sleep

The principal finding of the current investigation suggests that BIS values for flotation-REST are comparable to literature-derived results transpiring during relaxation-induction and stage I sleep. As well, the flotation-REST BIS values were substantially different compared to the awake state and stages II and III–IV sleep values. Additionally, BIS values for the awake state and sleep stages I, II, and III–IV were substantially variant from each other. According to standard sleep EEG criteria, wakefulness is characterized by > 50% of the epoch consisting of alpha activity in a relaxed subject with eyes closed or by < 50% of the epoch with low-voltage, mixed frequency (2–7 Hz) activity [35, 36]. Further, wakefulness is associated with an obvious presence of beta activity, especially with open eyes and while concentrating [37, 38]. However, stage I sleep is characterized by 50% of the epoch consisting of relatively low-voltage, mixed frequency (2–7 Hz) activity and < 50% of epoch containing alpha activity [35, 36]. Stage I sleep is largely defined by exclusion: a low-voltage, mixed frequency back ground EEG activity devoid of sleep spindles and K-complexes, cessation of blinking, absence of saccadic eye movements, and alpha activity in < 50% of the epoch [36]. Since evidence indicates that stage I sleep can also be quantitatively described according to alpha or theta dominance [39] or depicted by several unique qualitative EEG findings [40], it is clear that stage I sleep consists of a diverse array of electrophysiological subpopulations.

Although the precise electrophysiological basis for computing BIS values is unclear, certain publications have been elucidative [41, 42]. Evidence indicates that for BIS values 60–100, there is a near-perfect correlation between the BIS value and beta ratio (spectral power of 30–47 Hz ÷ power of 11–20 Hz) [43]. It is relevant that spectral entropy, an EEG processing technique similar to BIS monitoring, has been recently shown to discriminate the transition from the awake state to stage I sleep [44].

In the current investigation, BIS values progressively decreased from the awake state to stage I sleep, stage II sleep, and stages III–IV sleep. The delta plus theta amplitude has been shown to account for 50% of the relative power in the awake state [38]. During stage I sleep, when compared to the awake state, investigators have demonstrated an increase in relative delta plus theta power and a decrease in relative beta power [5, 38]. Further, it has been shown that relative to stage I sleep and when advancing from stage II to stage IV sleep, beta relative power progressively decreases, and theta plus delta relative power increases [5]; Uchida et al. demonstrated a power density reciprocal relationship between beta and delta activity across all stages of sleep [45]. Together, these observations provide an electrophysiological rationale as to reasons why BIS monitoring would discriminately correlate with the awake state and the various stages of sleep.

Several observations have been made during flotation-REST that are important; specifically, investigators have demonstrated that delta power [3] and theta power [4] increase during flotation-REST. Further, stage I and stage II sleep, using polysomnography, each have been found to account for 40% of the time during flotation-REST [3]. Additionally, Fine et al. concluded that the EEG during flotation-REST was consistent with stage I sleep [4].

### Cognitive experiences in stage I sleep and flotation-REST

Multiple functional changes have been described during stage I sleep: slow-rolling eye movements, drowsiness, decreased eyelid blinking, slow eye movements, increasing number of wrong answers to questions, and a decrease in response to audible tones [35]. Drowsiness, a manifestation of stage I sleep, is characterized by increased eyelid closure (decreased eyelid opening) and a propensity for decreased alertness and inclination to fall asleep [44, 46, 47]. Cognitive experiences during stage I sleep have been described as a dream-like or half-awake, half-asleep state and may include random visual images, thoughts, or feelings; a decrease in awareness of the surrounding environment; and an alteration in time awareness [40, 48]. Qualitatively comparable hypnagogic mental phenomena have also been found during flotation-REST, suggesting that the two states have similar experiential features [49, 50]. Vivid and detailed written documentations [51] and results from comprehensive questionnaires [49] of persons immediately following emersion from the flotation-REST tank indicate that some degree of awareness is present during floating. As some degree of vigilance has also been described during stage I sleep [48], these findings further suggest that stage I sleep and flotation-REST are likely to be relatively similar states.

### Physiology in stage I sleep and flotation-REST

Multiple physiologic alterations have been described during stage I sleep: decreased minute ventilation, muscle tone, heart rate, blood pressure, metabolic rate, and frontal lobe blood flow [35, 37]. Other investigations have demonstrated that flotation-REST is associated with reductions in blood pressure [52, 53] and levels of plasma cortisol [52, 54, 55], plasma adrenocorticotropic hormone [54], thyroid stimulating hormone [55], plasma noradrenalin [56], and urinary catecholamine [55]. Each of these five researchers concluded in their manuscript that flotation-REST is conducive to relaxation. Although physiological changes in flotation-REST do not circumscribe a specific sleep stage, it is plausible that stage I sleep is included in this array of physiological alterations.

### Relaxation induction and flotation-REST

In Hudetz et al.’s investigation of relaxing guided imagery, anxiety scores and BIS values decreased, indicating that a relaxation effect had indeed occurred [16]. The nadir BIS values with relaxation induction are virtually identical to the mean BIS values transpiring during the two flotation-REST sessions. This suggests that flotation-REST has an electrophysiological signature comparable to that of induced relaxation. BIS nadir onsets for the two relaxation-induction activities were at 15 and 55 min; however, during flotation-REST, the BIS value was substantially reduced within a couple of minutes, when compared to that in the awake state. This potentially speaks to the notion that flotation-REST may have a rapid onset and persistent calming effect. Since the relaxation-induction BIS values are similar to the stage I sleep results, this suggests that a peaceful demeanor is likely a feature of stage I sleep.

### Mood enhancement

The first author’s experience demonstrated that pre-flotation-REST mood scores increased in the later sessions compared to the early sessions. Further, the post-flotation-REST mood scores were significantly greater in contrast to the pre-flotation scores. These observations are corroborated by other flotation-REST studies that have demonstrated an objective improvement in relaxation [2, 18, 53, 55] and mood enhancement [18, 52].

## Conclusions

The objective BIS electrophysiological signature implies that relaxation-induction, stage I sleep, and flotation-REST may be comparable conditions of consciousness. The qualitative similarities regarding cognitive experiences and partial awareness that occur during stage I sleep and flotation-REST also suggest that the two are likely to be analogous states.

## Limitations

The principal limitation of this investigation is that only one person underwent flotation-REST while BIS monitoring was performed.

## Additional files

**Additional file 1.** Awake state BIS values. Data of awake state Bispectral Index™ values from previous studies.

**Additional file 2.** BIS values during relaxation-induction. Data of Bispectral Index™ values during relaxation-induction from previous studies.

## Abbreviations

BIS: Bispectral Index™
EEG: electroencephalography
REST: restricted environmental stimulation technique

## References

[1] Kjellgren A, Westman J (2014). Beneficial effects of treatment with sensory isolation in flotation-tank as a preventive health-care intervention—a randomized controlled pilot trial. BMC Complement Altern Med.

[2] Jonsson K, Kjellgren A (2016). Promising effects of treatment with flotation-REST (restricted environmental stimulation technique) as an intervention for generalized anxiety disorder (GAD): a randomized controlled pilot trial. BMC Complement Altern Med.

[3] Sakata S, Shinohara J, Hori T, Sugimoto S (1995). Enhancement of randomness by flotation rest (restricted environmental stimulation technique). Percept Mot Skills.

[4] Fine T, Mills D, Turner JW: Differential effects of wet and dry flotation REST on EEG frequency and amplitude. In: Barabasz AF, Barabasz M, editors. Clinical and experimental restricted environmental stimulation. New York: Springer; 1993. P. 205–213.

[5] Armitage R (1995). The distribution of EEG frequencies in REM and NREM sleep stages in healthy young adults. Sleep.

[6] Iwata K, Yamamoto M, Nakao M, Kimura M (1999). A study on polysomnographic observations and subjective experiences under sensory deprivation. Psychiatry Clin Neurosci.

[7] Iwata K, Nakao M, Yamamoto M, Kimura M (2001). Quantitative characteristics of alpha and theta EEG activities during sensory deprivation. Psychiatry Clin Neurosci.

[8] Suedfeld P, Steel GD, Wallbaum ABC, Bluck S, Livesey N, Capozzi L (1994). Explaining the effects of stimulus restriction: testing the dynamic hemispheric asymmetry hypothesis. J Environ Psychol.

[9] Dunham CM, Ransom KJ, McAuley CE, Gruber BS, Mangalat D, Flowers LL (2006). Severe brain injury ICU outcomes are associated with Cranial-Arterial Pressure Index and noninvasive Bispectral Index and transcranial oxygen saturation: a prospective, preliminary study. Crit Care.

[10] Dunham CM, Katradis DA, Williams MD (2009). The bispectral index, a useful adjunct for the timely diagnosis of brain death in the comatose trauma patient. Am J Surg.

[11] Benini F, Trapanotto M, Sartori S, Capretta A, Gobber D, Boniver C, Zacchello F (2005). Analysis of the bispectral index during natural sleep in children. Anesth Analg.

[12] Dahaba AA, Xue JX, Xu GX, Liu QH, Metzler H (2011). Bilateral Bispectral Index (BIS)-Vista as a measure of physiologic sleep in sleep-deprived anesthesiologists. Minerva Anestesiol.

[13] Tung A, Lynch JP, Roizen MF (2002). Use of the BIS monitor to detect onset of naturally occurring sleep. J Clin Monit Comput.

[14] Nieuwenhuijs D, Coleman EL, Douglas NJ, Drummond GB, Dahan A (2002). Bispectral index values and spectral edge frequency at different stages of physiologic sleep. Anesth Analg.

[15] Sleigh JW, Andrzejowski J, Steyn-Ross A, Steyn-Ross M (1999). The bispectral index: a measure of depth of sleep?. Anesth Analg.

[16] Hudetz JA, Hudetz AG, Reddy DM (2004). Effect of relaxation on working memory and the Bispectral Index of the EEG. Psychol Rep.

[17] Tsutsumi M, Nogaki H, Shimizu Y, Stone TE, Kobayashi T (2017). Individual reactions to viewing preferred video representations of the natural environment: a comparison of mental and physical reactions. Jpn J Nurs Sci.

[18] Driller MW, Argus CK (2016). Flotation restricted environmental stimulation therapy and napping on mood state and muscle soreness in elite athletes: a novel recovery strategy?. Perform Enhanc Health.

[19] Kaskinoro K, Maksimow A, Langsjo J, Aantaa R, Jaaskelainen S, Kaisti K, Sarkela M, Scheinin H (2011). Wide inter-individual variability of Bispectral Index and spectral entropy at loss of consciousness during increasing concentrations of dexmedetomidine, propofol, and sevoflurane. Br J Anaesth.

[20] Schuller PJ, Newell S, Strickland PA, Barry JJ (2015). Response of Bispectral Index to neuromuscular block in awake volunteers. Br J Anaesth.

[21] Miner JR, Biros MH, Seigel T, Ross K (2005). The utility of the Bispectral Index in procedural sedation with propofol in the emergency department. Acad Emerg Med.

[22] Liu J, Singh H, White PF (1997). Electroencephalographic Bispectral Index correlates with intraoperative recall and depth of propofol-induced sedation. Anesth Analg.

[23] Zhong T, Guo QL, Pang YD, Peng LF, Li CL (2005). Comparative evaluation of the cerebral state index and the Bispectral Index during target-controlled infusion of propofol. Br J Anaesth.

[24] Liu J, Singh H, White PF (1996). Electroencephalogram bispectral analysis predicts the depth of midazolam-induced sedation. Anesthesiology.

[25] Mi WD, Sakai T, Singh H, Kudo T, Kudo M, Matsuki A (1999). Hypnotic endpoints vs. the Bispectral Index, 95% spectral edge frequency and median frequency during propofol infusion with or without fentanyl. Eur J Anaesthesiol.

[26] Cortinez LI, Delfino AE, Fuentes R, Munoz HR (2007). Performance of the cerebral state index during increasing levels of propofol anesthesia: a comparison with the Bispectral Index. Anesth Analg.

[27] da Costa VV, Torres RVSD, Pires EC, Saraiva RA (2007). Comparison of the Bispectral Index in awake patients with cerebral palsy. Rev Bras Anestesiol.

[28] Fassoulaki A, Paraskeva A, Patris K, Pourgiezi T, Kostopanagiotou G (2003). Pressure applied on the extra 1 acupuncture point reduces Bispectral Index values and stress in volunteers. Anesth Analg.

[29] Fassoulaki A, Paraskeva A, Kostopanagiotou G, Tsakalozou E, Markantonis S (2007). Acupressure on the extra 1 acupoint: the effect on Bispectral Index, serum melatonin, plasma beta-endorphin, and stress. Anesth Analg.

[30] Valkenburg AJ, de Leeuw TG, Tibboel D, Weber F (2009). Lower Bispectral Index values in children who are intellectually disabled. Anesth Analg.

[31] Yeo J, Jeon Y (2015). Effects of stellate ganglion block on sedation as assessed by Bispectral Index in normal healthy volunteers. Pain Physician.

[32] Litscher G (2004). Effects of acupressure, manual acupuncture and Laserneedle acupuncture on EEG Bispectral Index and spectral edge frequency in healthy volunteers. Eur J Anaesthesiol.

[33] Kabukcu HK, Karakas MS, Yanikoglu A, Sahin N, Kabukcu M (2014). Use of Bispectral Index monitoring for determination of sedation depth in 50 patients undergoing cardioversion. J Pak Med Assoc.

[34] Yousef GT, Elsayed KM (2013). A clinical comparison of ketofol (ketamine and propofol admixture) versus propofol as an induction agent on quality of laryngeal mask airway insertion and hemodynamic stability in children. Anesth Essays Res.

[35] Silber MH, Ancoli-Israel S, Bonnet MH, Chokroverty S, Grigg-Damberger MM, Hirshkowitz M, Kapen S, Keenan SA, Kryger MH, Penzel T (2007). The visual scoring of sleep in adults. J Clin Sleep Med.

[36] Keenan S, Hirshkowitz M, Kryger MH, Roth T, Dement WC (2011). Monitoring and staging human sleep. Principles and practice of sleep medicine.

[37] Purves D. In: *Neuroscience.* 2nd edn. Edited by Purves D, Augustine GJ, Fitzpatrick D, Katz LC, LaMantia A-S, McNamara JO, Williams SM. Sunderland: Sinauer Associates, Inc.; 2001: 603–623.

[38] Morisson F, Lavigne G, Petit D, Nielsen T, Malo J, Montplaisir J (1998). Spectral analysis of wakefulness and REM sleep EEG in patients with sleep apnoea syndrome. Eur Respir J.

[39] Gora J, Colrain IM, Trinder J (1999). Respiratory-related evoked potentials during the transition from alpha to theta EEG activity in stage 1 NREM sleep. J Sleep Res.

[40] Goupil L, Bekinschtein TA (2012). Cognitive processing during the transition to sleep. Arch Ital Biol.

[41] Sigl JC, Chamoun NG (1994). An introduction to bispectral analysis for the electroencephalogram. J Clin Monit.

[42] Rampil IJ, Kim JS, Lenhardt R, Negishi C, Sessler DI (1998). Bispectral EEG index during nitrous oxide administration. Anesthesiology.

[43] Morimoto Y, Hagihira S, Koizumi Y, Ishida K, Matsumoto M, Sakabe T (2004). The relationship between Bispectral Index and electroencephalographic parameters during isoflurane anesthesia. Anesth Analg.

[44] Sriraam N, Padma Shri TK, Maheshwari U (2016). Recognition of wake-sleep stage 1 multichannel eeg patterns using spectral entropy features for drowsiness detection. Australas Phys Eng Sci Med.

[45] Uchida S, Maloney T, Feinberg I (1992). Beta (20–28 Hz) and delta (0.3–3 Hz) EEGs oscillate reciprocally across NREM and REM sleep. Sleep.

[46] Poudel GR, Innes CR, Jones RD (2012). Cerebral perfusion differences between drowsy and nondrowsy individuals after acute sleep restriction. Sleep.

[47] Wilkinson VE, Jackson ML, Westlake J, Stevens B, Barnes M, Swann P, Rajaratnam SM, Howard ME (2013). The accuracy of eyelid movement parameters for drowsiness detection. J Clin Sleep Med.

[48] Yang CM, Han HY, Yang MH, Su WC, Lane T (2010). What subjective experiences determine the perception of falling asleep during sleep onset period?. Conscious Cogn.

[49] Kjellgren A, Lyden F, Norlander T. Sensory isolation in flotation tanks: Altered states of consciousness and effects on well-being. In: the Qualitative Report. vol. 13; 2008: 636–656. URL: http://www.nova.edu/ssss/QR/QR13-4/kjellgren.pdf. Accessed on 08 Jan 2017.

[50] Norlander T, Kjellgren A, Archer T (2001). The experience of flotation-REST as a function of setting and previous experience of altered state of consciousness. Imagin Cognit Personal.

[51] Lilly JC, Lilly JC (1977). Tank logs: Experiences. The deep self.

[52] Turner JW, Fine T, Ewy G, Sershon P, Freundlich T (1989). The presence or absence of light during flotation restricted environmental stimulation: effects on plasma cortisol, blood pressure, and mood. Biofeedback Self Regul.

[53] Jacobs GD, Heilbronner RL, Stanley JM (1984). The effects of short term flotation REST on relaxation: a controlled study. Health Psychol.

[54] Turner JW, Fine TH (1983). Effects of relaxation associated with brief restricted environmental stimulation therapy (REST) on plasma cortisol, ACTH, and LH. Biofeedback Self Regul.

[55] Schulz P, Kaspar CH (1994). Neuroendocrine and psychological effects of restricted environmental stimulation technique in a flotation tank. Biol Psychol.

[56] Kjellgren A, Sundequist U, Norlander T, Archer T (2001). Effects of flotation-REST on muscle tension pain. Pain Res Manag.

