# Supplementary material for: Comparison of Bispectral Index™ values during the flotation restricted environmental stimulation technique and results for stage I sleep: a prospective pilot investigation
Source: BMC Res Notes. 2017 Nov 29;10:640. doi: 10.1186/s13104-017-2947-4 (PMC5707909; doi:10.1186/s13104-017-2947-4)
Supplement: Supplementary file 1 — Additional file 1. Awake state BIS values. Data of awake state Bispectral Index™ values from previous studies. [file 13104_2017_2947_MOESM1_ESM.doc]

**Additional file 1** Awake state BIS values

| **Study** | **Participants** | **BIS** |  | **Study** | **Participants** | **BIS** |
| --- | --- | --- | --- | --- | --- | --- |
| Kaskinoro, 2011 | 10 | 94.0 |  | Hudetz, 2004 | 42 | 96.0 |
| Schuller, 2015 | 10 | 94.0 |  | da Costa, 2007 | 113 | 96.6 |
| Miner, 2005 | 100 | 94.4 |  | Fassoulaki, 2003 | 25 | 97.0 |
| Liu, 1997 | 10 | 94.5 |  | Fassoulaki, 2007 | 12 | 97.0 |
| Kaskinoro, 2011 | 10 | 94.6 |  | Nieuwenhuijs, 2002 | 10 | 97.0 |
| Zhong, 2005 | 20 | 95.3 |  | Valkenburg, 2009 | 35 | 97.0 |
| Liu, 1996 | 26 | 95.4 |  | Yeo, 2015 | 20 | 97.0 |
| Mi, 1999 | 42 | 95.5 |  | Litscher, 2004 | 25 | 97.4 |
| Cortinez, 2007 | 15 | 95.6 |  | Kabukcu, 2014 | 50 | 97.6 |
| Kaskinoro, 2011 | 10 | 95.7 |  | Yousef, 2013 | 100 | 100 |
|  |  |  |  |  | **685** | **96.6 ± 1.7** |

BIS: Bispectral Index™
