# Supplementary material for: Comparison of Bispectral Index™ values during the flotation restricted environmental stimulation technique and results for stage I sleep: a prospective pilot investigation
Source: BMC Res Notes. 2017 Nov 29;10:640. doi: 10.1186/s13104-017-2947-4 (PMC5707909; doi:10.1186/s13104-017-2947-4)
Supplement: Supplementary file 2 — Additional file 2. BIS values during relaxation-induction. Data of Bispectral Index™ values during relaxation-induction from previous studies. [file 13104_2017_2947_MOESM2_ESM.doc]

**Additional file 2** BIS values during relaxation-induction

| **Study** | **Participants** | **Nadir BIS** | **Nadir Onset** | **Technique** |
| --- | --- | --- | --- | --- |
| Hudetz, 2004 | 42 | 88.9 | 15 minutes | guided imagery |
| Tsutsumi, 2017 | 12 | 80.0 | 55 minutes | videos |
|  | **54** | **82.8 ± 6.8** |  |  |

BIS: Bispectral Index™
